# Circular RNA ZNF609 promotes the malignant progression of glioma by regulating miR-1224-3p/PLK1 signaling

**DOI:** 10.7150/jca.54934

**Published:** 2021-04-12

**Authors:** Senjie Du, Hongying Li, Fen Lu, Shang Zhang, Jian Tang

**Affiliations:** Department of Rehabilitation, Children's Hospital of Nanjing Medical University, Jiangsu, China.

**Keywords:** circular RNA ZNF609, glioma, miRNA sponge, malignant progression

## Abstract

**Objective:** Previous studies have demonstrated that circular RNAs (circRNAs) play vital roles in pathological process of various diseases, including tumors. This study aimed at exploring the role and mechanism of circRNA RNA ZNF609 (circ-ZNF609) in the occurrence and development of glioma.

**Materials and methods:** Real-time quantitative PCR (qRT-PCR) was applied to measure the expression of circ-ZNF609, miRNA-1224-3p (miR-1224-3p) and Polo-like kinase 1 (PLK1) in glioma tissues and cell lines. Furthermore, the association between circ-ZNF609 and clinical features of glioma was analyzed. CCK8 assay, EdU assay and Transwell assay were conducted to detect the effect of circ-ZNF609, miR-1224-3p and PLK1 on proliferation, migration and invasion in glioma cells. Then, we investigated the underlying mechanism of circ-ZNF609 by bioinformatics analysis, luciferase reporter assay, RNA immunoprecipitation (RIP), qRT-PCR and western blotting assay.

**Results:** Circ-ZNF609 was confirmed prominently upregulated in glioma. Inhibition of circ-ZNF609 could obviously suppress glioma cell proliferation, migration and invasion, while overexpression of circ-ZNF609 promoted glioma growth and metastasis. *In vivo*, xenotransplanted tumor model also showed that overexpression of circ-ZNF609 could promote *in vivo* glioma growth. Mechanistically, circ-ZNF609 could promote PLK1 expression via binding to miR-1224-3p, circ-ZNF609/miR-1224-3p/PLK1 was shown responsible for circ-ZNF609 promoting glioma growth and metastasis.

**Conclusion:** Together, our results revealed that circ-ZNF609 elevates glioma growth and metastasis via enforcing PLK1 expression by competitively binding miR-1224-3p, suggesting that circ-ZNF609 might be an underlying therapeutic target for glioma.

## Introduction

Glioma is the most prevalent type of primary nervous system tumors with a poor prognosis 1-3. Glioblastoma is the most aggressive type of glioma and the median overall survival of it is within 15 months. Incidence and mortality of glioma appear to be increasing rapidly in the past few decades as a result of this highly metastatic disease 4, 5. Although the recent treatments like targeted biologic therapy and adjuvant radiotherapy have been markedly improved, overall patient survival is not satisfactory as a result of local recurrence and distant metastasis 6, 7. Thus, identification of potential pathogenic mechanisms of glioma and therapeutic approaches against glioma are significantly indispensable.

Circular RNAs (circRNAs) is a novel member of noncoding RNAs (ncRNAs). Different from linear RNAs, circRNAs are characterized by free 5'- or 3'-ends connection with more stable structure 8, 9. Although advanced achievements have been made in recent years, the underlying regulatory mechanism of many circRNAs remain to be further explored. Moreover, circRNAs have been regarded as a “molecular sponge” to promote the expression of the downstream genes via harboring multiple microRNAs (miRNAs) and affect their activity therefore 10, 11. Emerging evidence demonstrates that circRNAs have attracted more attention in various diseases by regulating many pathological and physiological process 12-14, including cell growth, cell cycle, apoptosis, metastasis and differentiation. For examples, circRNA hsa_circ_0072995 promoted the progression of epithelial ovarian cancer (EOC) via modulating miR-147a/CDK6 axis^15^; has_circ_0000140 could restrain the cell proliferation, migration, invasion and glycolysis metabolism of oral squamous cell carcinoma (OSCC) by promoting CDC73 expression via binding to miR-182-5p^16^; circRNA hsa_circ_0002577 has been verified to be upregulated in endometrial cancer and it might accelerate cancer progression by activating IGF1R/PI3K/Akt pathway 17. However, the potential biological roles of circRNAs in glioma remain to be further explored.

Circ-ZNF609 (circBase ID: hsa_circ_0000615) is located in chromosome 15.64q and originated from ZNF609 18. ZNF609 is a member of zinc finger family, which is involved in regulation of gene expression 19. CircZNF609 has been reported to be dysregulated in many tumors such as renal carcinoma 20, lung adenocarcinoma 21, nasopharyngeal carcinoma 22, colorectal cancer 23, hepatocellular carcinoma 24, gastric cancer 25 and prostate cancer 26. Nevertheless, the biological roles of circZNF609 in glioma remain unclear. In this study, we verified a circRNA deriving from the ZNF609 gene locus, named circ-ZNF609 whose expression was increased in glioma. Besides, *in vitro* and *in vivo* assays demonstrated that circ-ZNF609 was positively interrelated to the growth and metastasis of glioma. Mechanistically, circ-ZNF609 might promote PLK1 expression by binding to miR-1224-3p, so as to promote glioma growth and metastasis. Hence, circ-ZNF609 might be a potential clinical biomarker and therapeutic target in glioma.

## Material and Methods

### GEPIA database

GEPIA is a new interactive website for TCGA- and GTEx-based analysis of RNA sequence data (http://gepiacancer-pku.cn). A total of 518 glioma tissues and 207 normal control tissues were obtained from TCGA database to analyze mRNA expression profile. ZNF609 expression in low grade glioma tissues and normal control tissues were analyzed.

### Clinical samples

62 glioma tissues and 15 non-glioma tissues were recruited from the Children's Hospital of Nanjing Medical University between January 2015 and October 2018. None of the patients had preoperative radiotherapy or chemotherapy. All the tissues were collected by surgery and confirmed by two independent pathologists. The tissue biopsies were stored at -80 °C. The informed consent forms were obtained from all participants and this study was approved by the Medical Ethics Committee of Children's Hospital of Nanjing Medical University.

### Cell culture and transfection

The glioma cell lines (LN229, LN308, U87 and U251) and HEB (human normal brain glial cells) were obtained from the Chinese Academy of Sciences. All the cells were cultivated in Dulbecco's modified Eagle's Medium (Gibco, USA) containing 10% fetal bovine serum (Gibco, USA) at 37 °C with 5% CO_2_.

For circ-ZNF609 overexpression, circ-ZNF609 overexpression plasmids were constructed into pSicoR lentiviral vector, and 293T cells were used for virus production. Lentiviruses were used to infect glioma cells and puromycin was used to select for two weeks to produce circ-ZNF609 overexpressed cell lines. U87 and U251 cells were placed in 6-well plates (5x10^4^ per well) and transfected with circ-ZNF609 siRNAs, miR-1224-3p mimics and inhibitor, PLK1 siRNAs and corresponding negative references by using Lipofectamine 3000 (Invitrogen, USA) for 24 h in accordance with the manufacturer's protocol. All the transfection reagents were generated by GenePharma (Shanghai, China).

### Quantitative real-time polymerase chain reaction (qRT-PCR)

Total RNA from glioma tissues and cell lines was extracted by utilizing TRIzol reagent (Invitrogen, USA) in accordance with the manufacturer's instructions. Then, reverse transcription was conducted to obtain cDNA. Subsequently, the qRT-PCR assay was conducted with SYBR Green PCR Master Mix (TakaRa, Japan) on LightCycler system (Roche, Basel, Switzerland). RNase R assay and Actinomycin D assay were utilized to verify the stability of circ-ZNF609 and linear ZNF609 mRNA expression level. The relative expression level was examined by the 2^-ΔΔCT^ method and GAPDH or U6 small nuclear RNA was used as internal controls. The primer sequences are shown in Table 1.

### Cell proliferation assay

CCK8 assay: After transfection, cells (4×10^3^) were transferred into a 96-well plate. After incubation for 24 h, each well was added with 10 µl of CCK-8 reagent for 2 h at 37 °C, after the indicated time (0, 24, 48, 72 and 96 h). Then, the OD values at 450 nm were examined by a microplate reader at 450 nm.

EdU assay: EdU kit (RiboBio, China) was utilized for the assay. Transfected glioma cells were placed in 96-well plate at 3000 cells/well, and then the culture medium was added with 50 mM EdU solution. 24 h later, cells were fixed in 4% formaldehyde and infiltrated using Triton X-100. Treated cells were incubated with EdU reagent and counterstained using Hoechst (Thermo Fisher, USA). The staining results under fluorescence microscope were calculated. Pictures of five fields of view randomly selected under microscope were taken to further analyze.

### Transwell assay

Transwell assay was conducted using 24-well plate with membrane filter chamber at aperture 8μm. For invasion assay, Matrigel™ (BD Biosciences) was precoated in the upper chamber. U87 and U251 cells were selected into the upper chamber at 5×10^4^/well by using 100 μL FBS-free DMEM culture medium, while 600 μl DMEM culture medium with 10% FBS were seeded into the lower chamber. The chambers were incubated at 37 °C for 24 h, the cells in upper chamber were removed by cotton swabs. Then the cells adhering to the under layer of the membranes were fixed into methanol and stained using crystal violet. Finally, a microscope was used to capture the images of cells. Transwell plates used for the migration assay did not have the upper chambers coated with Matrigel.

### Subcellular fractionation location

Briefly, we transferred glioma cells into EP tubes and added Lysis Buffer J for 20 min. After centrifugation, the obtained supernatant contained cytoplasmic RNA. Next, we incubated the remaining liquid with nuclear lysate solution (RIPA) for 20 min. After centrifugation, the obtained supernatant contained nuclear RNA. QRT-PCR assay was performed to verify the subcellular localization of circ-ZNF609.

### Luciferase assay

Circ-ZNF609 and PLK1 3'untranslated region (3'UTR) wild sequence and mutant sequence with or without miR-1224-3p binding sites were synthesized by Genepharm. According to the instructions of the manufacturer, Promega kit (Promega, USA) was employed for measuring luciferase activity as per the IFU and recorded.

### RNA immunoprecipitation

U87 or U251 cells were collected and lysed using Magna RIP Kit (EMD Millipore), and then incubated with protein G Sepharose beads (GE Healthcare) coated with anti-AGO2 antibody (Abcam) at 4 °C overnight, and anti-IgG antibody was used as the negative control. RNA was then isolated for qRT-PCR.

### Western blot

The proteins were extracted from glioma tissues or cell lines utilizing lysis buffer of protease inhibitor PMSF. The BCA Kit was applied to analyze the protein concentration. Then proteins (20 μg) were separated on SDS-PAGE gels and blotted on PVDF membrane. After blocking in 5% skim milk, the primary antibody was added for incubation. After cleaning the membrane, secondary antibody was incubated before exposure. Then the membrane incubated with primary antibodies targeting PLK1 (ab189139; Abcam, Cambridge, MA, USA) or GAPDH (1:1000; Beyotime, Nantong, China) overnight at 4 °C. Next, the membranes were washed using TBS for three times and incubated with an HRP-linked goat antirabbit secondary antibody (ab205718; Abcam) at room temperature for 2 h. The ECL Western Blotting Substrate Kit (ab65623; Abcam) was used to detect protein signals.

### Xenograft mouse models

After LV-circ-ZNF609-U87 cells and LV-NC-U87 cells were stably transfected, approximately 2 × 10^5^ stably transfected cells were injected subcutaneously into the right limbs of the e BALB/c nude mice. Tumor weights and volumes (volumes (mm^3^) = length × width^2^/2) were measured 30 days after subcutaneous injection. This study had gained approval of the ethics committee of Children's Hospital of Nanjing Medical University.

### Statistics analysis

All the results were expressed as mean ± SEM and analyzed with GraphPad Prism 8.0 software. Student's t-test was utilized for the comparison of between-group difference. The overall survival rate (OS) was analyzed by Kaplan-Meier method and the log-rank test. All the assays were repeated for three times. P < 0.05 was considered statistically significant.

## Results

### Circ-ZNF609 expression level was obviously elevated in glioma

**Figure 1:**
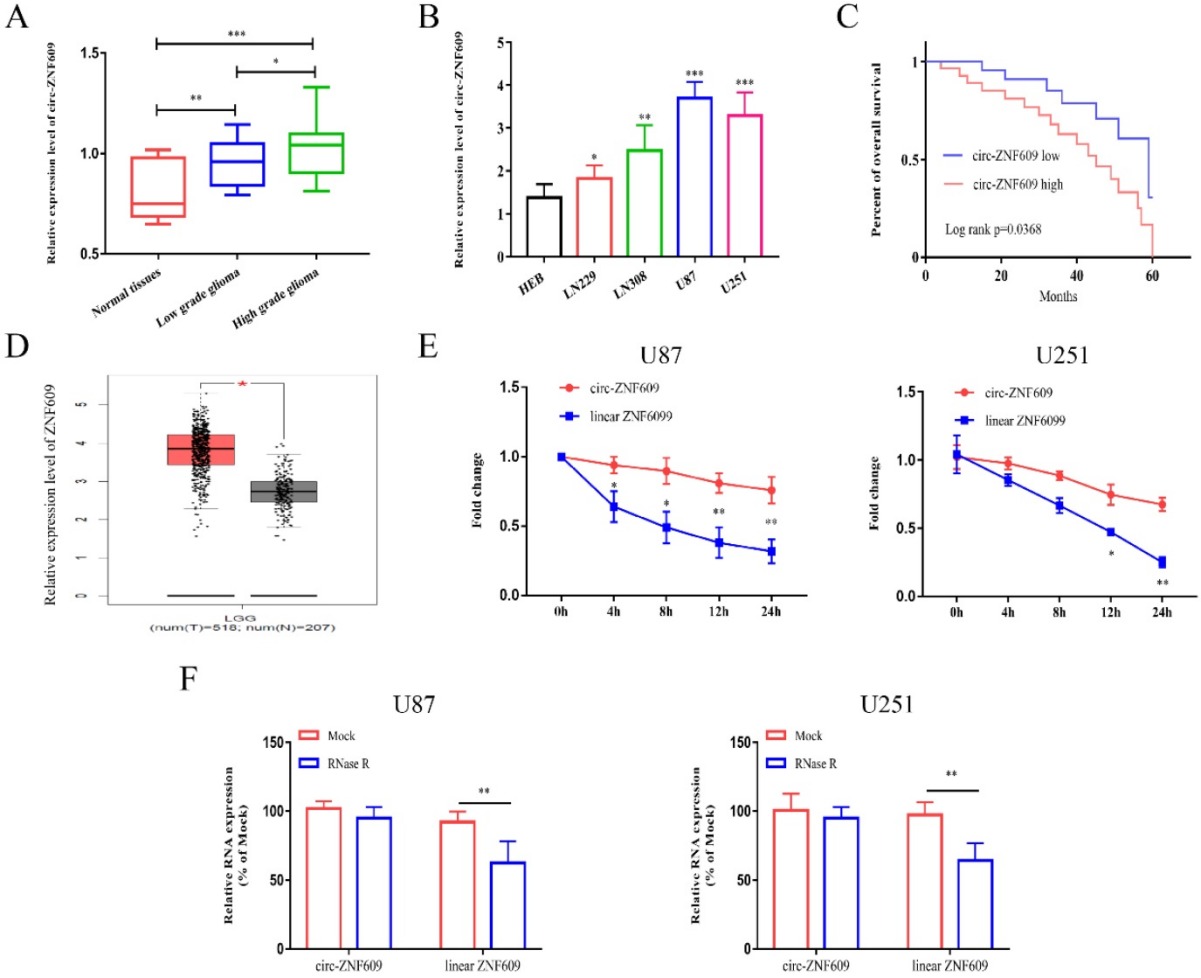
** Circ-ZNF609 expression level was obviously elevated in glioma.** (A) The expression level of circ-ZNF609 in glioma tissues was increased compared to expression in matched normal tissues. (B) Relative expression of circ-ZNF609 in glioma cell lines compared to expression in HEB cells. (C) Kaplan-Meier analysis of the overall survival of glioma patients with high and low expression of circ-ZNF609 (p=0.0368). (D) ZNF609 expression in low grade glioma tissues was analyzed by TCGA database. (E) U87 and U251 cells were treated with actinomycin D, the mRNA expression of circ-ZNF609 and ZNF609 was measured by qRT-PCR. (F) The stability of circ-ZNF609 was evaluated by RNase R test in U87 and U251 cells. *p<0.05; **p<0.01; ***p<0.001. All data are showed as mean ± SD. The experiment was conducted for three times.

Firstly, we detected the expression of circ-ZNF609 in glioma tissues and normal control tissues by conducting qRT-PCR assay. The results indicated that the level of circ-ZNF609 in glioma was markedly increased. Besides, the expression level of circ-ZNF609 in high grade glioma was higher than that in low grade glioma (Figure 1A). We measured the expression of circ-ZNF609 in glioma cell lines, and found that circ-ZNF609 expression was markedly increased in glioma cell lines compared to HEB (Figure 1B). After analyzing, we found that circ-ZNF609 expression was interrelated to the poor prognosis of glioma patients (Figure 1C). Furthermore, the results of GEPIA database indicated that linear ZNF609 was much higher in low grade glioma tissues than that in normal tissues (Figure 1D). Besides, to examine the stability of circ-ZNF609, the transcriptional inhibitor actinomycin D was used to co-culture with U87 and U251 cells and then detected the expression of circ-ZNF609 and linear ZNF609. The results indicated that the half-life of linear ZNF609 mRNA was less than 12 h, while that of circ-ZNF609 was more than 24 h (Figure 1E). Moreover, the results of RNase R experiment indicated that RNase R treatment could significantly reduce the mRNA expression level of ZNF609, while the expression level of circ-ZNF609 was not affected after RNase R treatment (Figure 1F).

### Inhibition of circ-ZNF609 suppressed the proliferation, migration and invasion of glioma cells

**Figure 2:**
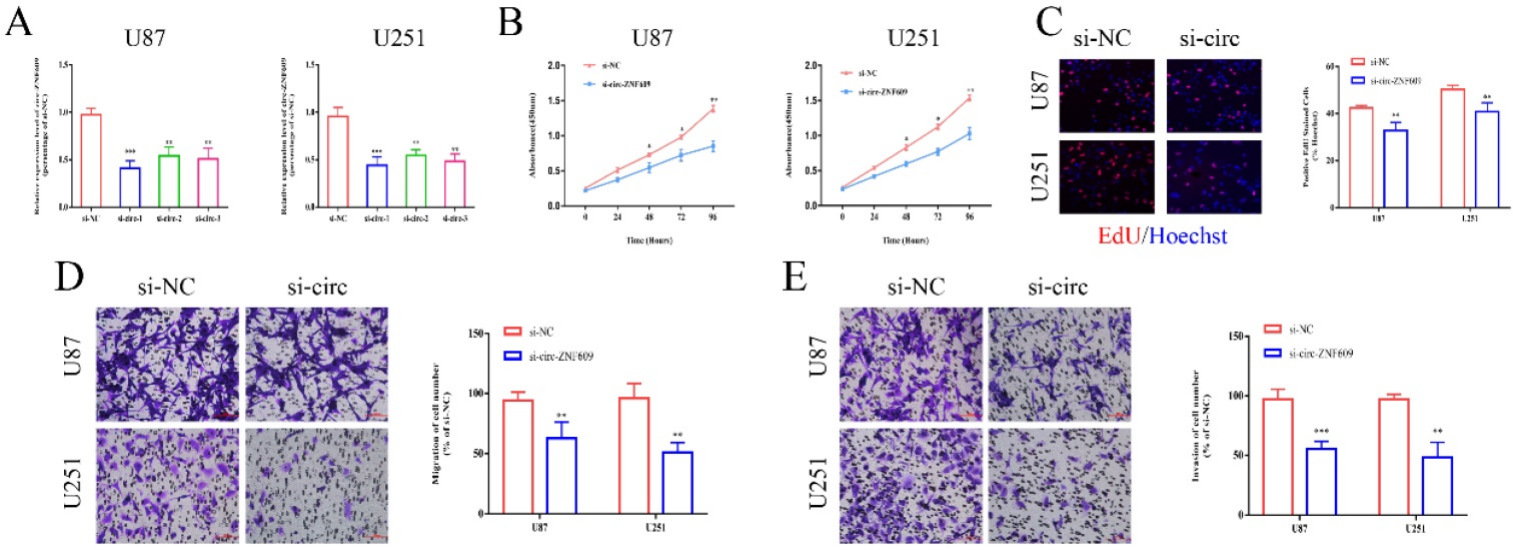
** Inhibition of circ-ZNF609 suppressed the proliferation, migration and invasion of glioma cells.** (A) Specific small interference RNA (siRNAs) was synthesized in accordance with circ-ZNF609 sequence and transfected into U87 and U251 cells. The interference efficiency was detected by qRT-PCR. (B-C) CCK8 assay and EdU assay were applied to measure the effect of circ-ZNF609 on the proliferation of U87 and U251 cells (magnification: 200x). The results showed that inhibition of circ-ZNF609 could suppress the proliferation of glioma cells. (D-E) The metastatic ability of U87 and U251 cells was examined by transwell migration and invasion assay after suppressing the expression of circ-ZNF609 in U87 and U251 cell lines (magnification: 200x). The results showed that inhibition of circ-ZNF609 could suppress the migration and invasion of glioma cells. *p<0.05; **p<0.01; ***p<0.001. All data are showed as mean ± SD. The experiment was conducted for three times.

To confirm the potential function of circ-ZNF609 in glioma, small interfering RNAs (siRNAs) were applied to interfere with the expression level of circ-ZNF609 in U87 and U251 cell lines. The results of qRT-PCR indicated that circ-ZNF609 siRNAs could effectively suppress the expression of circ-ZNF609, while the interference efficiency of siRNA-1 was higher than that of others (Figure 2A). Then, we detected the biological function of circ-ZNF609 on cell proliferation, migration and invasion in glioma cell lines. The results of CCK8 assay and EdU assay indicated that inhibition of circ-ZNF609 could markedly repress cell proliferation (Figure 2B-C). Moreover, transwell assay verified that knockdown of circ-ZNF609 could remarkedly suppress migration and invasion of glioma cell lines (Figure 2D-E). All above results proved that circ-ZNF609 could promote cell proliferation, migration and invasion in glioma.

### Circ-ZNF609 could bind to miR-1224-3p

**Figure 3:**
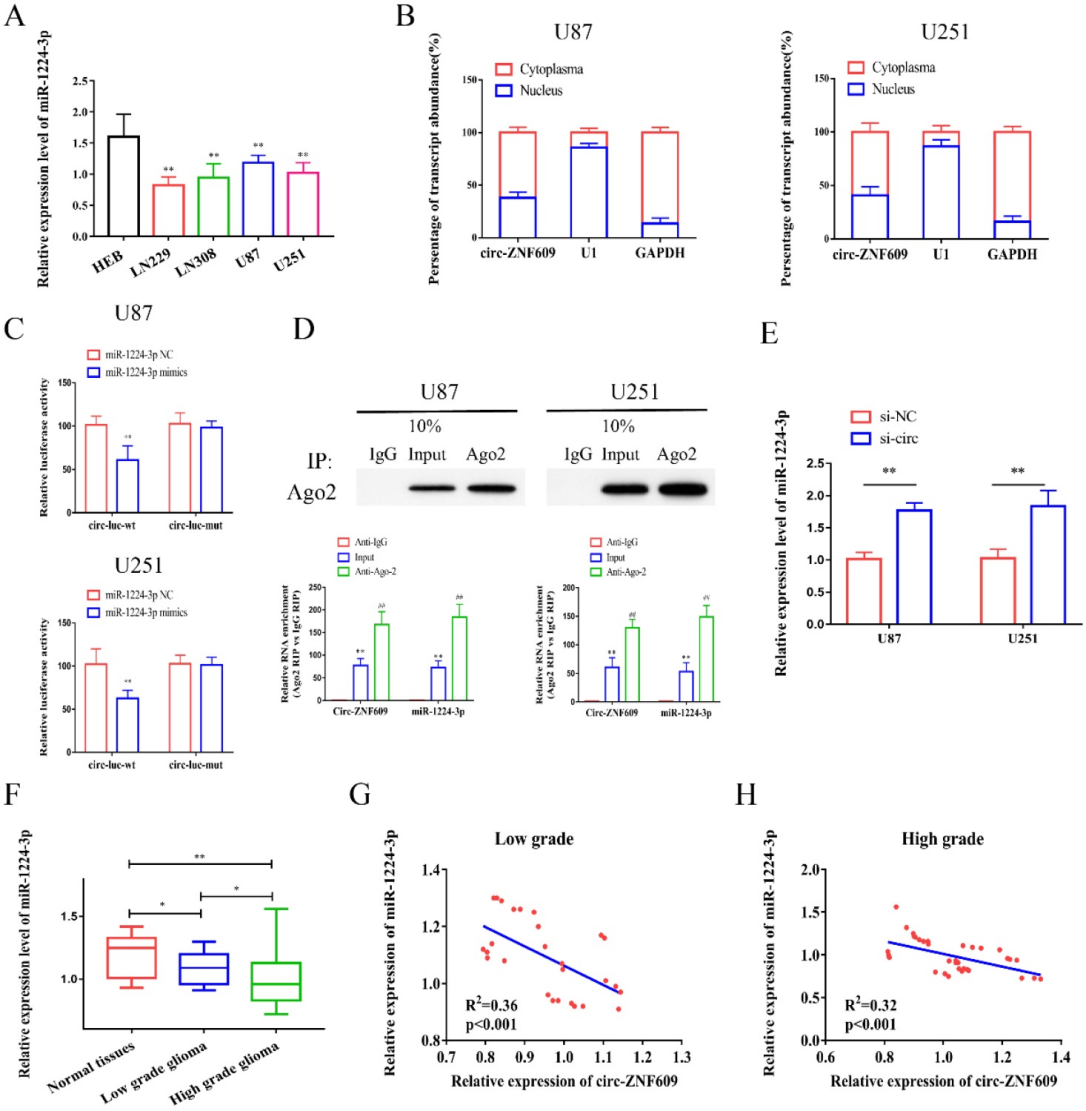
** Circ-ZNF609 could bind to miR-1224-3p.** (A) Relative expression of miR-1224-3p in glioma cell lines compared to expression in HEB cells. (B) Subcellular positioning of circ-ZNF609 in U87 and U251 cells through nuclear-cytoplasmic separation assay, the results showed that circ-ZNF609 is mainly located in the cytoplasm of glioma cell lines. (C) Relative luciferase activities were investigated in glioma cells after transfection with circ-ZNF609-WT or circ-ZNF609-Mut and miR-1224-3p mimic or miR-NC. (D) Enrichment of circ-ZNF609 and miR-1224-3p in Ago2-containing beads of U87 and U251 cells. (E) The expression of miR-1224-3p by qRT-PCR was detected after inhibition of circ-ZNF609 in U251 and U87 cells. (F) The expression of miR-1224-3p in glioma and adjacent tissues was tested by qRT-PCR, the results showed that the expression level of miR-1224-3p in glioma tissues was decreased compared to expression in matched normal tissues. (G-H) Through Person's correlation analysis, it was found that the expression of circ-ZNF6090 in low grade (R^2^=0.36, p<0.001) and high grade glioma tissue (R^2^=0.32, p<0.001) was significantly negatively correlated with miR-1224-3p expression.*p<0.05; **p<0.01; ^##^p<0.01. All data are showed as mean ± SD. The experiment was conducted for three times.

Emerging evidence has demonstrated that circRNAs could serve as ceRNAs and regulate downstream genes expression via sponging miRNAs. Hence, bioinformatics (https://circinteractome.nia.nih.gov/) was analyzed to detect the potential miRNAs might sponge circ-ZNF609, and miR-1224-3p possessed the highest bound fraction with circ-ZNF609 (Figure S1A). Then, we measured the expression of miR-1224-3p in glioma cell lines. The results indicated that the relative level of miR-1224-3p in glioma cell lines was significantly decreased (Figure 3A). Then, we conducted nuclear-cytoplasmic separation assay and identified that circ-ZNF609 might exist mainly in the cytoplasm of U87 and U251 cells, suggesting that circ-ZNF609 could participate in post-transcriptional regulation. Therefore, miR-1224-3p was chosen for further research. According to the predicted binding sites, we synthesized circ-ZNF609 wild-type plasmids (circ-wt) and mutant plasmids (circ-mut) (Figure S1B). Dual-luciferase reporter assay was performed to clarify the binding relationship between circ-ZNF609 and miR-1224-3p. As shown in Figure 3C, the results of luciferase reporter assay demonstrated that miR-1224-3p mimics remarkedly suppressed the luciferase activity of circ-ZNF609-wt reporter, suggesting that miR-1224-3p might sponge circ-ZNF609. RNA immunoprecipitation (RIP) assay showed that both circ-ZNF609 and miR-1224-3p were significantly enriched in Ago2-containing beads compared with IgG-containing beads or the input group (Figure 3D) Moreover, qRT-PCR revealed that circ-ZNF609 inhibition promoted miR-1224-3p expression (Figure 3E). Additionally, qRT-PCR indicated that miR-1224-3p expression was obviously decreased in glioma tissues (Figure 3F). Besides, correlation analysis verified that circ-ZNF609 expression was negatively interrelated to miR-1224-3p expression in low grade glioma tissues (R^2^=0.36, p<0.001) (Figure 3G) and high grade glioma tissues (R^2^=0.32, p<0.001) (Figure 3H).

### MiR-1224-3p served as a suppressor in the proliferation, migration, and invasion of glioma cells

**Figure 4:**
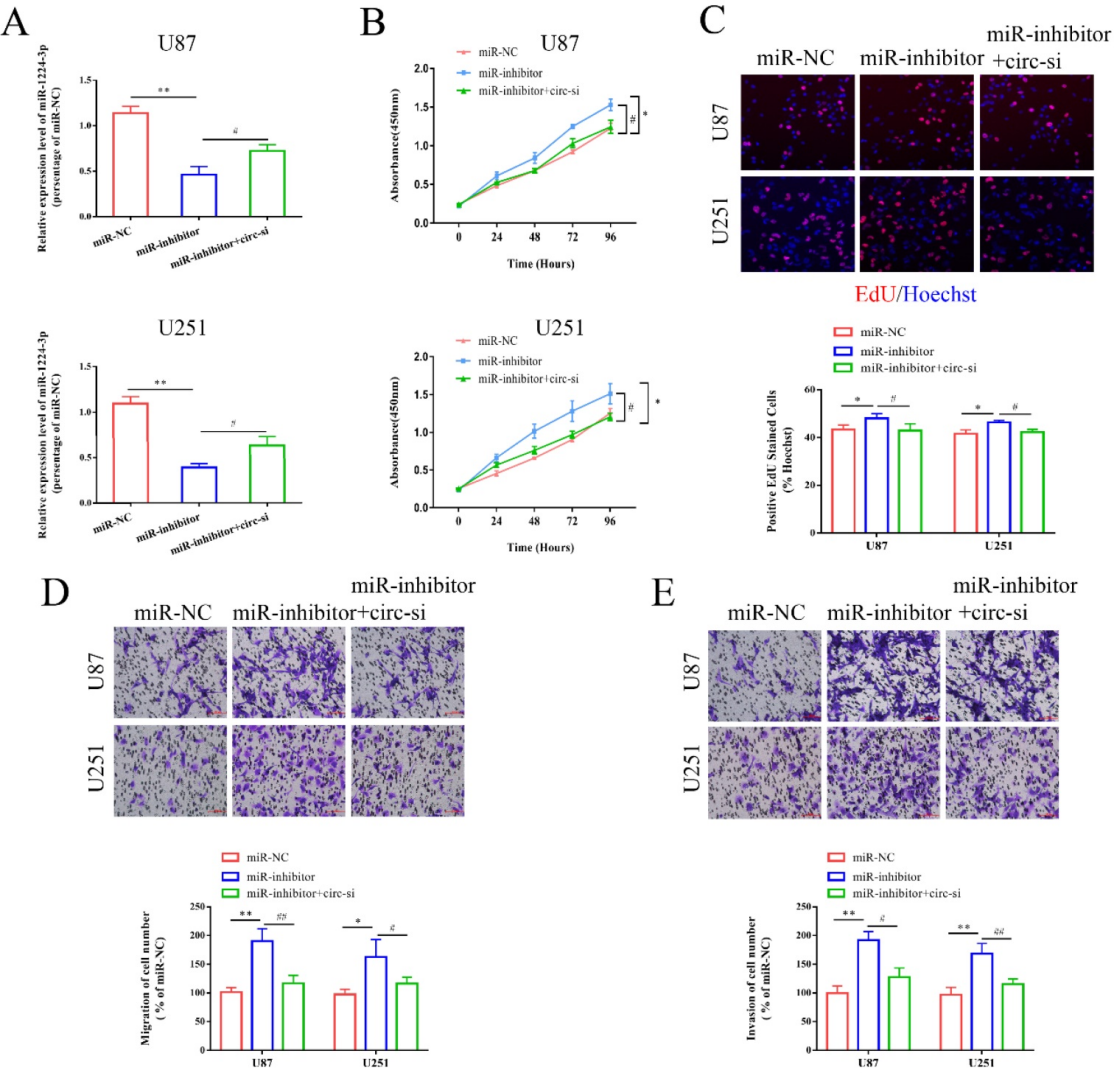
** MiR-1224-3p served as a suppressor in the proliferation, migration, and invasion of glioma cells.** (A) After inhibition of miR-1224-3p and circ-ZNF609 in U87 and U251 cells, the expression of miR-1224-3p was measured. (B-C) The proliferation of U87 and U251 cells were detected by CCK8 assay and EdU assay after suppressing miR-1224-3p and circ-ZNF609 simultaneously (magnification: 200x). (D-E) After inhibition of miR-1224-3p and circ-ZNF609 in U87 and U251 cells, the ability of cell migration and invasion was detected by Transwell assays (magnification: 200x). *p<0.05; **p<0.01;^ #^p<0.05; ^##^p<0.01. All data are showed as mean ± SD. The experiment was conducted for three times.

To confirm the functional interaction between circ-ZNF609 and miR-1224-3p in glioma cells, we conducted the rescue experiment using qRT-PCR assay, CCK8 assay, EdU assay and transwell assay. As shown in Figure 4A, the miR-1224-3p inhibitor significantly decreased the expression level of miR-1224-3p, while co-transfection of circ-ZNF609-si promoted miR-1224-3p expression level. The results of CCK8 assay and EdU assay demonstrated that transfection of miR-1224-3p inhibitor remarkedly promoted the proliferation of U87 and U251 cells, while circ-ZNF609 overexpression plasmid (circ-ZNF609-si) could relatively rescue the facilitating effect of miR-1224-3p inhibitor (Figure 4B, C). Furthermore, the results of transwell assay suggested that the inhibition in miR-1224-3p facilitated the migration and invasion of U87 and U251 cells, while inhibiting circ-ZNF609 expression level could reduce the facilitating effect of miR-1224-3p inhibitor (Figure 4D, E).

### PLK1 is a target of miR-1224-3p

**Figure 5:**
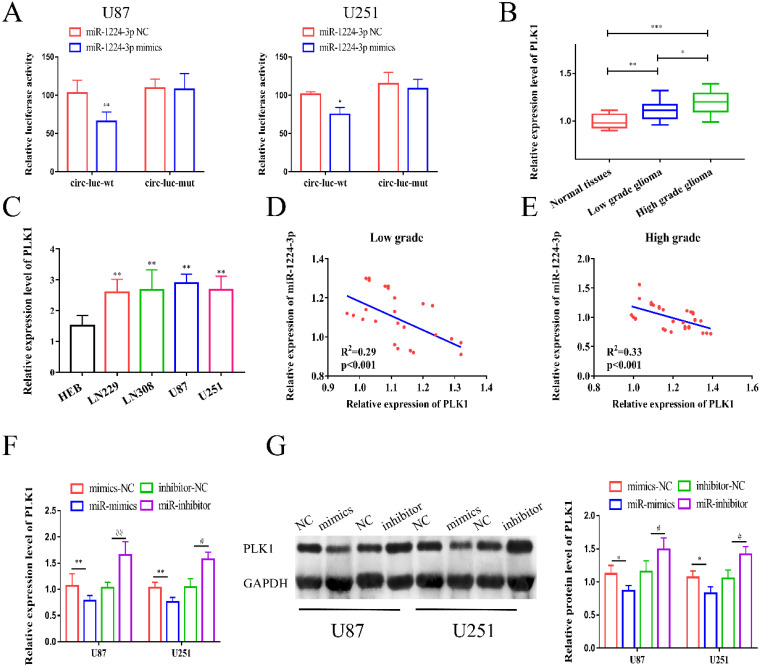
** PLK1 is a target of miR-1224-3p.** (A) Relative luciferase activities were investigated in glioma cells after transfection with PLK1-WT or PLK1-Mut and miR-1224-3p mimic or miR-NC. (B) The expression level of PLK1 in glioma tissues was increased compared to expression in matched normal tissues. (C) Relative expression of PLK1 in glioma cell lines compared to expression in HEB cells. (D-E) Through Person's correlation analysis, it was found that the expression of PLK1 in low grade (R^2^=0.29, p<0.001) and high grade glioma tissue (R^2^=0.33, p<0.001) was significantly negatively correlated with miR-1224-3p expression. (F-G) The mRNA and protein expression of PLK1 was detected after overexpression or inhibition of miR-1224-3p in U87 and U251 cells. *p<0.05; **p<0.01; ***p<0.001; ^#^p<0.05; ^##^p<0.01. All data are showed as mean ± SD. The experiment was conducted for three times.

Next, we explored the potential target genes of miR-1224-3p through RNAhybird, microRNA.org, TargetScan and miRDB, and we determined that PLK1 may be the downstream gene. Hence, we chose it for further study (Figure S1C). According to the predicted binding sites, we synthesized a PLK1 wild type plasmid (PLK1-wt) and a mutant plasmid (PLK1-mut) (Figure S1D). As shown in Figure 5A, the results of luciferase reporter assay showed that miR-1224-3p mimics markedly suppressed the luciferase activity of PLK1-wt reporter, suggesting that miR-1224-3p might target PLK1. Furthermore, we explored the expression level of PLK1 in glioma and found that PLK1 expression was extremely increased in glioma tissues and cell lines (Figure 5B,C). Then, we analyzed the correlation between miR-1224-3p and PLK1 in glioma tissues and a negative relationship between these two molecules was observed in low grade glioma tissues (R^2^=0.29, p<0.001) (Figure 5D) and high grade glioma tissues (R^2^=0.33, p<0.001) (Figure 5E). Moreover, after transfection of miR-1224-3p mimics and inhibitor in glioma cells, we examined the level of PLK1. We verified that the expression of PLK1 decreased both at mRNA and protein level after overexpressing miR-1224-3p, while suppressing miR-1224-3p could significantly elevate the expression level of PLK1 (Figure 5F, G). Overall, we concluded that circ-ZNF609 might participate in the progression of glioma through sponging miR-1224-3p to increase the expression level of PLK1.

### MiR-1224-3p might inhibit proliferation, migration and invasion of glioma cells through targeting PLK1

**Figure 6:**
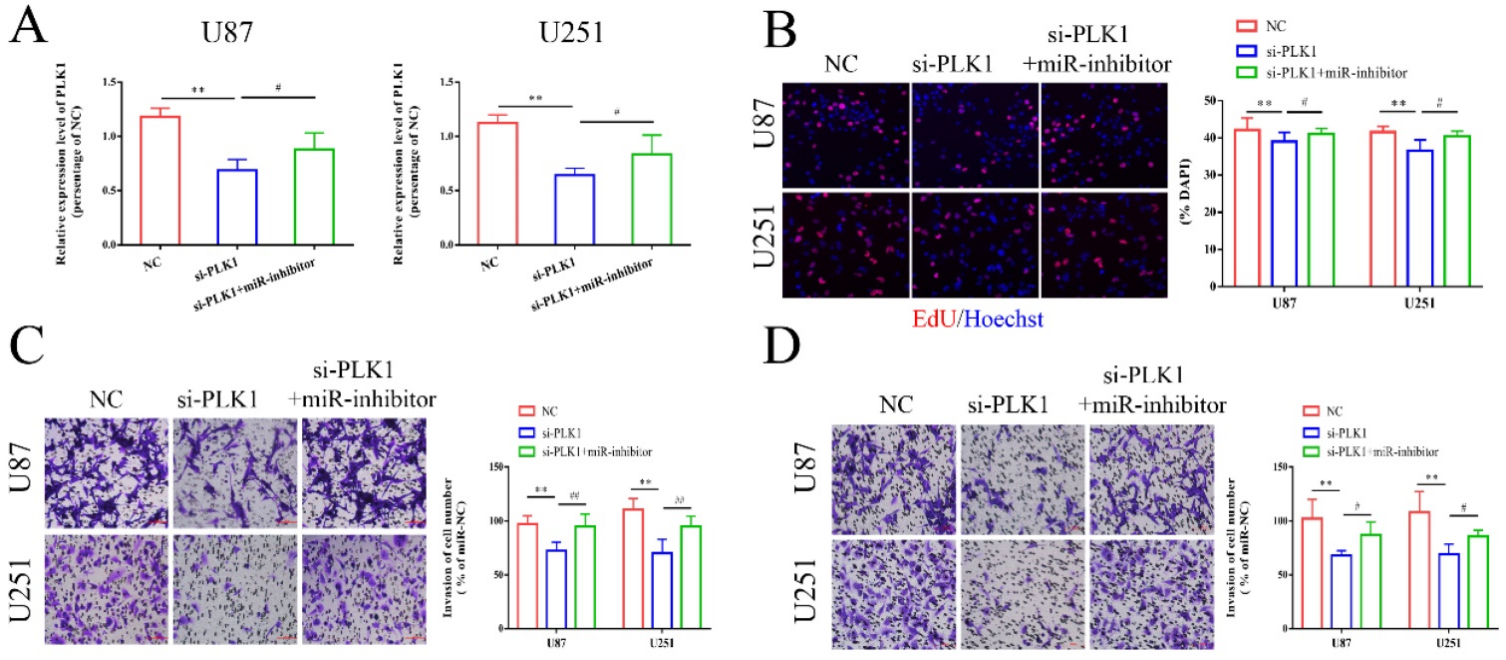
** MiR-1224-3p might inhibit proliferation, migration and invasion of glioma cells through targeting PLK1.** (A) PLK1-si and miR-1224-3p inhibitor were transfected into U87 and U251 cells, and the expression of PLK1 was measured. (B) After transfection of PLK1-si and miR-1224-3p inhibitor, EdU assay was performed to measure the cell proliferation of U87 and U251 cells (magnification: 200x). (C-D) After transfection of PLK1-si and miR-1224-3p inhibitor, Transwell assay was performed to measure the cell migration and invasion of U87 and U251 cells (magnification: 200x). **p<0.01; ^#^p<0.05; ^##^p<0.01. All data are showed as mean ± SD. The experiment was conducted for three times.

Next, we co-transfected glioma cells with PLK1 siRNA and miR-1224-3p inhibitor, and examined the expression level of PLK1. The results revealed that the level of PLK1 in the si-PLK1 group was remarkedly decreased, while the expression level of PLK1 in the si-PLK1+miR-1224-3p inhibitor group were obviously higher than that in the si-PLK1 group (Figure 6A). Then, we conducted *in vitro* experiments to explore the influence of PLK1 and miR-1224-3p on glioma cell. As shown in Figure 6B-D, we identified that decreased expression level of PLK1 in glioma cells obviously inhibited cell proliferation, migration, and invasion, while co-transfection of PLK1 siRNA and miR-1224-3p inhibitor in glioma cells respectively reversed the inhibition influence of si-PLK1 on glioma cells. This suggested that miR-1224-3p may inhibit proliferation, migration and invasion of glioma cells by targeting PLK1.

### Circ-ZNF609 might regulate PLK1 expression by binding to miR-1224-3p

**Figure 7:**
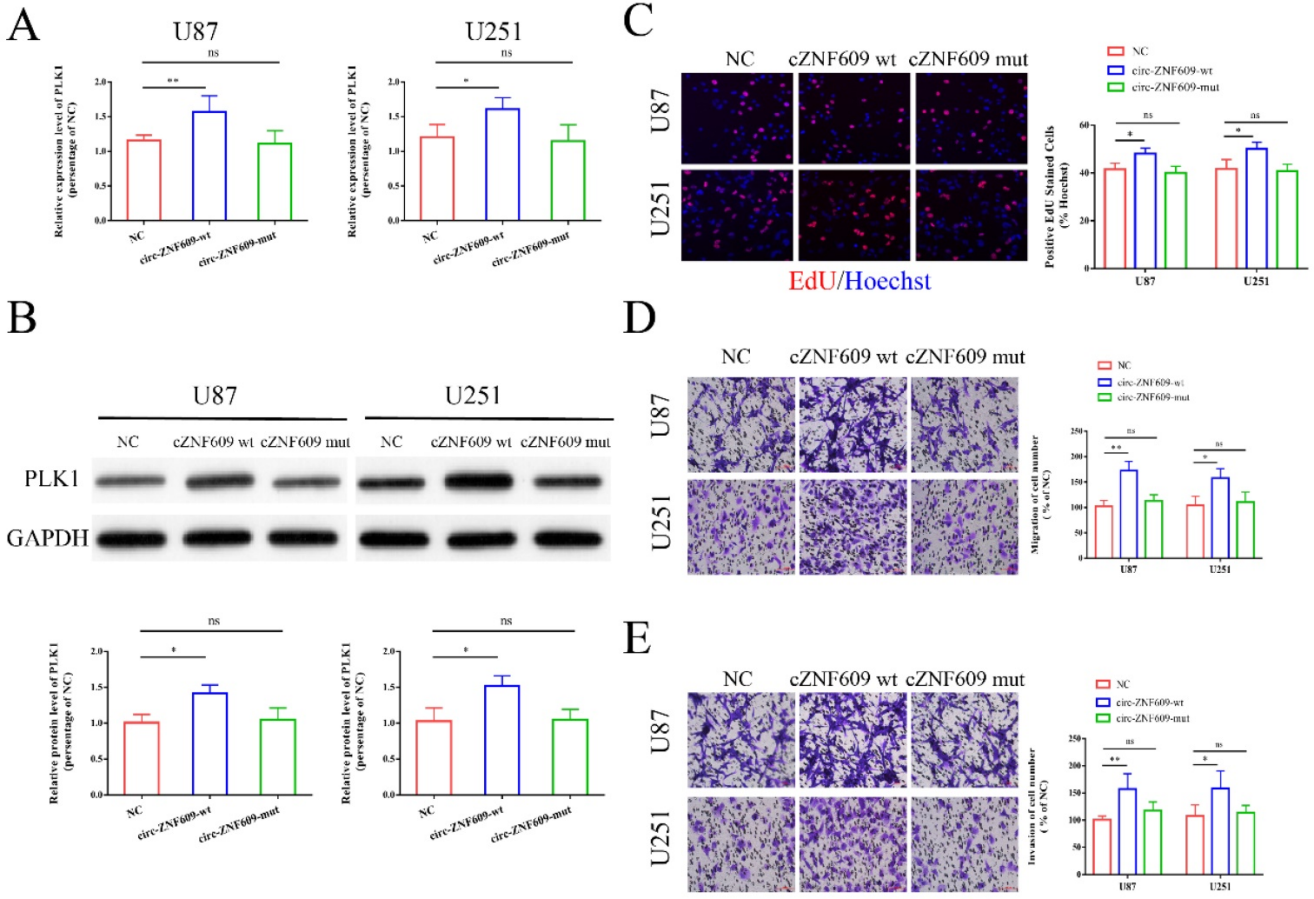
** Circ-ZNF609 might regulate PLK1 expression by binding to miR-1224-3p.** (A-B) circ-ZNF609-wt and circ-ZNF609-mut were transfected into U87 and U251 cells, and the mRNA and protein expression level of PLK1 was measured by qRT-PCR and western blot. (C) After transfection of circ-ZNF609-wt and circ-ZNF609-mut, EdU assay was performed to measure the cell proliferation of U87 and U251 cells, the results showed that circ-ZNF609-wt could promote the proliferation of glioma cells while circ-ZNF609-mut could not affect the proliferation of cells (magnification: 200x). (D-E) After transfection of circ-ZNF609-wt and circ-ZNF609-mut, Transwell assay was performed to measure the cell migration and invasion of U87 and U251 cells, the results showed that circ-ZNF609-wt could promote the migration and invasion of glioma cells while circ-ZNF609-mut could not affect the proliferation of cells (magnification: 200x); * p<0.05; **p<0.01; ns, no significant difference. All data are showed as mean ± SD. The experiment was conducted for three times.

After transfection of U87 and U251 cells with circ-ZNF609 wild type plasmid (circ-ZNF609-wt) and circ-ZNF609 mutant type plasmid (circ-ZNF609-mut), we examined the mRNA and protein expression level of PLK1 and found that PLK1 in the circ-ZNF609-wt group was remarkedly increased, while the no obvious change was observed in the circ-ZNF609-mut group (Figure 7A, B). Then, we conducted experiments to explore the effects of circ-ZNF609-wt and circ-ZNF609-mut on glioma cell. As shown in Figure 7C-E, we found that transfection of circ-ZNF609-wt in glioma cells obviously promoted cell proliferation, migration, and invasion, while transfection of circ-ZNF609-mut could not affect the cell function of glioma cells. These results suggested that circ-ZNF609 might regulate PLK1 expression through sponging miR-1224-3p.

### Upregulation of circ-ZNF609 promoted the growth of glioma *in vivo*

**Figure 8:**
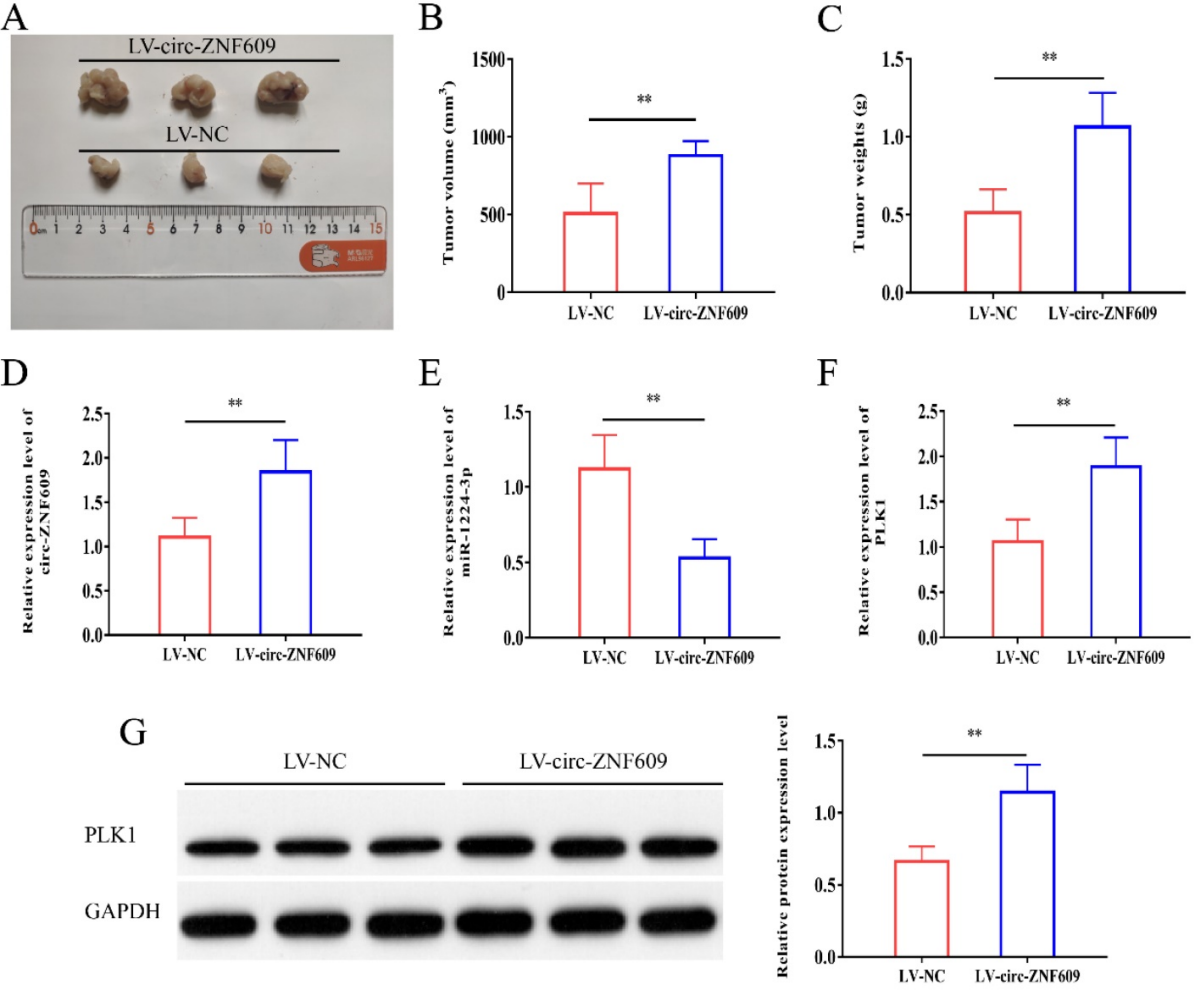
** Upregulation of circ-ZNF609 promoted the growth of glioma *in vivo.***(A) Representative images of neoplasms from each group of nude mice. (B) Determination of tumor volumes. (C) Determination of tumor weights. (D) The qRT-PCR showed that circ-ZNF609 expression was incresed in tumor tissues of the LV- circ-ZNF609 group. (E) The qRT-PCR results indicated that expression of miR-1224-3p was decreased in tumor tissues of the LV-circ-ZNF609 group. F-G. QRT-PCR and western blot results indicated that circ-ZNF609 overexpression promoted PLK1 expression, **p<0.01. All data are showed as mean ± SD. The experiment was carried out for three times.

We constructed stably overexpressed circ-ZNF609 or LV-NC U87 cells for the formation of tumors in immunodeficient mice to further confirm the function of circ-ZNF609 *in vivo*. As shown in Figure 8A-C, overexpression of circ-ZNF609 contributed to increases in tumor volume and weight *in vivo*. Furthermore, the results of qRT-PCR revealed that circ-ZNF609 and PLK1 expression were increased in the tumor tissues of the LV‐circ-ZNF609 group, while miR‐1224-3p expression was significantly decreased (Figure 8D-G). Overall, elevated expression of circ-ZNF609 markedly promoted glioma growth *in vivo*.

## Discussion

Glioma is a prevalent intracranial malignancy contributes to considerable cancer-related mortality 6, 27. Meanwhile, it is characterized by insensitive to conventional chemo-radiotherapy 28, 29. CircRNA is a class of noncoding RNA generated from a non-canonical back splicing process, from a covalent bond between 5' and 3' ends of a single-stranded RNA 30-32.

Recent years, many cancer-associated circRNAs have been identified and verified as potential clinical biomarkers and therapeutic targets in various types of tumors, including glioma. For examples, Zheng et al. found that circular RNA TTBK2 (circ-TTBK2) could promote glioma malignancy via modulating miR-217/HNF1β/Derlin-1 pathway 33; Chen et al. revealed that the expression of circPTN was markedly elevated in glioma, circPTN might act as a tumour promoter through sponging miR-145-5p/miR-330-5p 34; Cao et al. showed that hsa_circ_0037251 could function as an oncogenic gene via a hsa_circ_0037251/miR-1224-3p/mTOR axis in glioma, and these potential biomarkers might be therapeutic targets 35. However, the precise roles of circRNAs in human diseases remain to be further explored.

Firstly, we identified that circ-ZNF609 expression was significantly elevated in glioma tissues and cell lines by qRT-PCR assay. Besides, functional assays demonstrated that overexpression of circ-ZNF609 could markedly promote the proliferation, migration and invasion of glioma cells. Emerging evidence revealed that circRNAs might act as a ceRNA to modulate the expression of downstream genes via functioning as miRNA sponge on the whole progression of various types of diseases. As is known to us, miRNAs have the ability to serve as independent regulators in various types of biological progression. To explore the potential mechanism of circ-ZNF609, nuclear-cytoplasmic separation assay was performed and we found that circ-ZNF609 mainly existed in the cytoplasm of glioma cells, which meant that circ-ZNF609 might serve as a ceRNA to bind to miRNA, so as to regulate the expression of downstream genes. Then, we found that several miRNAs might bind to circ-ZNF609. On basis of biological function and bound fraction, we speculated that miR-1224-3p might be a potential binding target. MiR-1224-3p has been reported to be downregulated in lung adenocarcinoma and could suppress cell growth and metastasis 21. Subsequently, the results of functional experiments demonstrated that circ-ZNF609 might sponge miR-1224-3p and inhibit its expression. Besides, miR-1224-3p could significantly inhibit the growth and metastasis of glioma cells. MiR-1224-3p expression was also decreased in glioma.

It has been identified that miRNAs might modulate gene expression via targeting downstream genes 3'UTR. Based on bioinformatics analysis, we found that PLK1 might be the potential target of miR-1224-3p. Polo-like kinases (PLKs) are a family of serine/threonine protein kinases which are widespread in eukaryotic cells, while PLK1 was the most investigated 36, 37. PLK1 plays multiple roles in the biological progression, including cell division, cell cycle, cell growth and metastasis 38, 39. PLK1 has been verified to be involved in various cancers, such as hepatocellular carcinoma 40, breast cancer 41, prostate cancer 42, acute myeloid leukemia 43. Additionally, PLK1 has been reported to be upregulated in glioma and can markedly promote cell proliferation and migration 44-46. Moreover, we demonstrated that circ-ZNF609 could promote the expression of PLK1 via sponge miR-1224-3p. Functional experiments identified that overexpression of PLK1 promoted the cell proliferation, migration and invasion in glioma.

In our findings, we suggest that circ-ZNF609 might significantly promote the proliferation, migration and invasion of glioma cells through the miR-1224-3p/PLK1 axis, whose function might involve molecular targets beneficial for diagnosing and treating glioma. Meanwhile, there are several parts remain to be explored in the further study. First, we need to conduct more experiments to verify other biological effects of circ-ZNF609. Second, the other potential regulatory effect of circ-ZNF609 on related pathway proteins remain to be further explored.

## Conclusion

In conclusion, our study suggests that circ-ZNF609 might act as an oncogene to promote glioma progression via regulating miR-1224-3p/PLK1 axis. Therefore, circ-ZNF609 might potentially be applied in the future diagnosis and treatment in glioma.

## Supplementary Material

Supplementary figure S1.

## Figures and Tables

**Table 1:** Sequences of primers for qRT-PCR

Name	Sequence
**Circ-ZNF609**	
Forward	5'- TGAGTGTCGCCTGCTAAAGA-3'
Reverse	5'- CCCCCAGCTTTCCTATTTTC-3'
**miR-1224-3p**	
Forward	5'- GAGGACTCGGGAGGTGGAG-3'
Reverse	5'- CTGAGGAGAGAGGAGGTGGG-3'
**PLK1**	
Forward	5'- AAAGAGATCCCGGAGGTCCTA-3'
Reverse	5'- GGCTGCGGTGAATGGATATTTC-3'
**GAPDH**	
Forward	5'- TGCACCACCAACTGCTTAGC-3'
Reverse	5'- GGCATGGACTGTGGTCATGAG-3'
**U6**	
Forward	5'- CGCTTCGGCAGCACATATAC-3'
Reverse	5'- TTCACGAATTTGCGTGTCAT-3'
