# Supplementary material for: Circular RNA ZNF609 promotes the malignant progression of glioma by regulating miR-1224-3p/PLK1 signaling
Source: J Cancer. 2021 Apr 12;12(11):3354–66. doi: 10.7150/jca.54934 (PMC8100806; doi:10.7150/jca.54934)
Supplement: Supplementary file 1 — Supplementary figure S1. [file jcav12p3354s1.pdf]

A

CircRNA Mirbase ID	CircRNA (Top) - miRNA (Bottom) pairing	Site Type	CircRNA Start	CircRNA End	3' pairing	local AU	position	TA	SPS	context+ score	context+ score percentile
hsa_circ_0000615 (5' ... 3')<br>hsa-miR-1224-3p (3' ... 5')	GAGCCUUGAAUCUUGAGGUGGGA<br>||||||| GACUCCUCUCUCCUCCACCCC	8mer-1a	107	114	0.003	0.057	-0.087	0.038	-0.137	-0.373	99

B

circ-ZNF609 wt 5’-GAGCCUUGAAUCUUGAGGUGGGA-3’

miR-1224-3p 3’- GACUCCUCUCUCCUCCACCCC-5’

circ-ZNF609 mut 5’-GAGCCUUGAAUCUUGUCCACCCA-3’

C

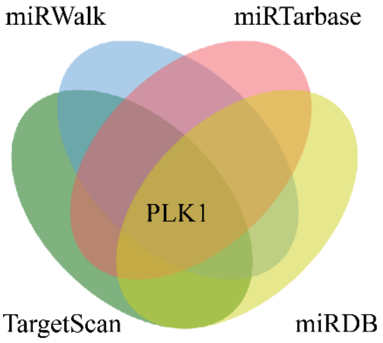

D

PLK1 wt 5’-CUGCAUCAUCCUUGCAGGUGGGG-3’

miR-1224-3p 3’-GACUCCUCUCUCCUCCACCCC-5’

PLK1 mut 5’-CUGCAUCAUCCUUGCUCCACCCG-3’

Fig S1: Circ-ZNF609 might act as a ceRNA to bind to miR-1224-3p with PLK1

(A) Bioinformatics websites (cirinteractome: https://circinteractome.nia.nih.gov/) was used to predict miRNAs that could bind to circ-ZNF609. (B) The bioinformatics website predicted the binding sites of circ-ZNF609 and miR-1224-3p, and constructed circ-ZNF609 wild-type plasmids and mutant plasmids. (C) Possible targets of miR-1224-3p were predicted through the bioinformatics website (miRWalk, miRTarbase, TargetScan and miRDB) and PLK1 may be a potential target through the intersection. (D) The bioinformatics website predicted the binding sites of PLK1 and miR-1224-3p, and constructed PLK1 wild-type plasmids and mutant plasmids.
